# Finite-Element Investigation of the Structural Behavior of Basalt Fiber Reinforced Polymer (BFRP)- Reinforced Self-Compacting Concrete (SCC) Decks Slabs in Thompson Bridge

**DOI:** 10.3390/polym10060678

**Published:** 2018-06-18

**Authors:** Lingzhu Zhou, Yu Zheng, Susan E. Taylor

**Affiliations:** 1 School of Environment and Civil Engineering, Dongguan University of Technology, Dongguan 523080, China; lingzhu_zhou@163.com; 2 Key Laboratory of Coastal and Offshore Engineering, Dalian University of Technology, Dalian 116024, China; 3 School of Natural and Built Environment, Queen’s University Belfast, Northern Ireland, BT9 5AG, UK; s.e.taylor@qub.ac.uk

**Keywords:** bridge deck slabs, SCC, BFRP, CMA, finite element analysis, structural behavior

## Abstract

The need for a sustainable development and improved whole life performance of concrete infrastructure has led to the requirement of more durable and sustainable concrete bridges alongside accurate predictive analysis tools. Using the combination of Self-Compacting Concrete (SCC) with industrial by-products and fiber-reinforced polymer (FRP), reinforcement is anticipated to address the concerns of high carbon footprint and corrosion in traditional steel-reinforced concrete structures. This paper presents a numerical investigation of the structural behavior of basalt fiber-reinforced polymer (BFRP)-reinforced SCC deck slabs in a real bridge, named Thompson Bridge, constructed in Northern Ireland, U.K. A non-linear finite element (FE) model is proposed by using ABAQUS 6.10 in this study, which is aimed at extending the previous investigation of the field test in Thompson Bridge. The results of this field test were used to validate the accuracy of the proposed finite element model. The results showed good agreement between the test results and the numerical results; more importantly, the compressive membrane action (CMA) inside the slabs could be well demonstrated by this FE model. Subsequently, a series of parametric studies was conducted to investigate the influence of different parameters on the structural performance of the deck slabs in Thompson Bridge. The results of the analyses are discussed, and conclusions on the behavior of the SCC deck slabs reinforced by BFRP bars are presented.

## 1. Introduction

It has been widely recognized that the durability of concrete bridge deck slabs is reduced as a consequence of the corrosion of the reinforcements [1,2,3]. Because of their excellent mechanical and non-corrosion properties, fiber-reinforced polymer (FRP) bars have been used in concrete bridge deck slabs as a replacement of conventional steel reinforcement, which aims to solve the problem of corrosion in internal steel reinforcement and improve the service life of the bridges [4,5,6,7,8,9]. However, compared to steel reinforcement, the lower elastic modulus of FRP reinforcements results in FRP-reinforced concrete bending members exhibiting greater deformation than steel-reinforced bending members [8]. The mechanical mechanism and design control index of FRP-reinforced bridge deck slabs were studied, with the aim to promote the use of FRP bars for actual bridge deck slabs.

**Figure 1:**
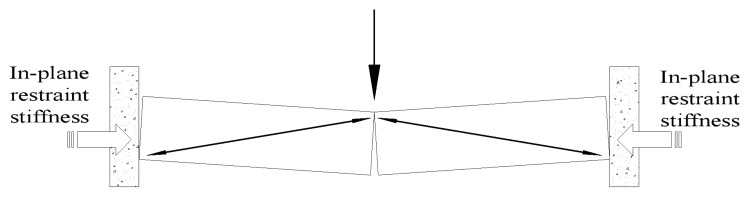
Compressive membrane action (CMA).

In an existing bridge, the bridge deck slabs of a typical beam–slab composite bridge have inherent strength provided by the lateral restraint of beam-to-slab boundary conditions. Bridge deck slabs subjected to wheel load are restrained against horizontal expansion because of the presence of an inherent in-plane restraint, which is the compressive membrane action (CMA) or arching action (see Figure 1) [10,11]. Research by Kirkpatrick et al. [12] has shown that the CMA had a beneficial effect on the serviceability limit state of bridge deck slabs, and current crack control formulas are not applicable to slab-on-girder bridge deck slabs. Additionally, it is reported in the literature by Zheng et al. [8,13] that the punching effects became more significant because of the increase of the CMA, and current design standards are highly conservative in predicting the ultimate bearing capacity of bridge deck slabs. The calculation method and mechanism of force transmission of slab-on-girder bridge deck slabs are not well understood, which led to more research work on the structural performance of concrete bridge deck slabs.

Currently, to improve the whole life performance of concrete infrastructures and drive sustainable development, the traditional reinforced concrete structures are planned to be replaced by sustainable construction with adequate material by means of design optimization. Self-Compacting Concrete (SCC) with an additional mineral provides an engineering material which has attracted wide attention from researchers and engineers because of its advantages [14,15,16]. Post-wastes materials, such as ground granulated blast furnace slag (GGBS), fly ash (FA), and limestone powder (LSP), are used in SCC to improve workability and flowability. SCC is environment-friendly and low-energy concrete because the cement is replaced by mineral additions, and, simultaneously, SCC is a high-performance concrete that can spread into place under its own weight without vibration and can make the construction of heavily congested structural elements easier [17,18].

Until now, the investigation of FRP-reinforced concrete using SCC in construction has been widely developed. However, the study of FRP-reinforced SCC structures, particularly in concrete deck slabs, is significantly limited. To investigate the feasibility of using BFRP-reinforced SCC structures in the construction industry, we carried out a field test and a study on SCC deck slabs reinforced with BFRP bars in a real bridge, named Thompson Bridge, in Northern Ireland, U.K. [19]. Because of the high cost of the experimental test and the low accuracy in predicting the ultimate state of the concrete deck slabs by the current design code, the finite element method (FEM) was adopted to comprehensively analyze the structural behavior of Thompson Bridge deck slabs, particularly emphasizing the effect of the arching action.

The aim of this paper was to study the behavior of SCC deck slabs with BFRP reinforcing bars under the wheel load. To extend our understanding of the ultimate state and failure mechanisms of Thompson Bridge deck slabs, a numerical investigation through the 3D finite element model was used in this paper [8,20,21]. It was shown that the numerical results of the 3D finite element model displayed a good agreement with the test results. Subsequently, the effect of CMA, ultimate bearing capacity, failure mechanisms, and stress distribution in different phases of BFRP-reinforced SCC deck slabs were presented. Finally, some parametric studies were conducted: (a) reinforcement ratio; (b) reinforcement type; (c) concrete compressive strength; (d) depth of the bridge deck slabs.

## 2. Experimental Program

### 2.1. Introduction of Thompson Bridge

**Figure 2:**
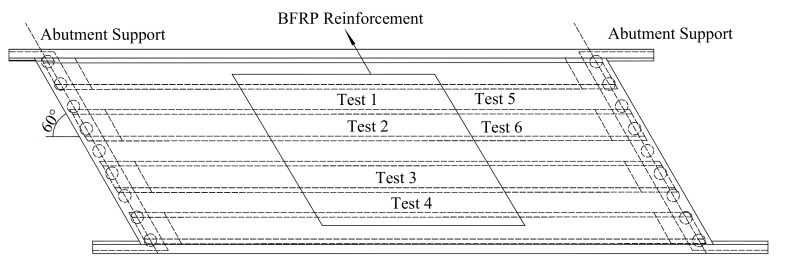
Test area of Thompson Bridge.

**Figure 3:**
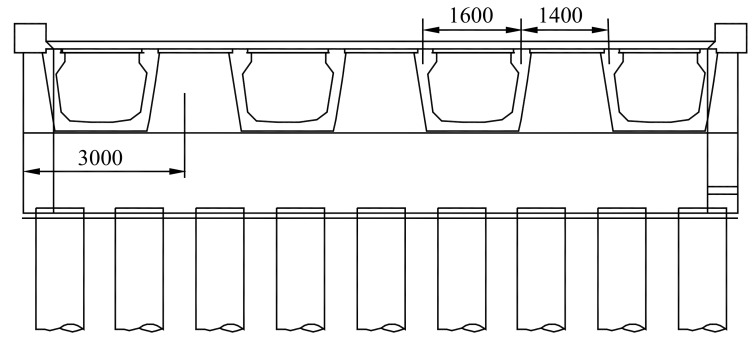
Profile of Thompson Bridge.

Thompson Bridge is a replacement bridge on the A509 trunk road in County Fermanagh, Northern Ireland. The length and width of Thompson Bridge are 32 and 11.85 m, respectively, and the thickness of the entire deck slab is 200 mm [19]. It consists of a fully integral single-span skew bridge with four W-beams supporting a SCC deck slab (see Figure 2 and Figure 3). Thompson Bridge is a modern short to medium span bridge deck used in Europe and the rest of the world. As shown in Figure 1, the mid-span section of Thompson Bridge slab was constructed with BFRP bars of 12 mm diameter, and the remaining slab had 12 mm steel reinforcement. This project aimed to assess the influence of the SCC and reinforcement type on the bridge deck slab. The reinforcement ratio of the bridge deck slab in the central region reinforced by BFRP is 0.6%, and the remaining slab reinforced by steel is 0.7%. The effective span of Thompson Bridge slab between W-beams and W-beams is 1400 mm, and that between the W-beams is 1600 mm (see Figure 3).

### 2.2. Material Properties

Table 1 summarizes the work performance and compressive strength of SCC based on rheological tests carried out on site at the time of casting and cube samples. Portland Cement (PC) of class 42.5 and limestone powder (LSP) were used in the self-compacting concrete of Thompson Bridge slab, and the cement used complied with Standard BS EN 197-1 CEM II. The SCC mixture proportion was designed by the minimum cement content of Roads Service allowed, and Superplasticizer (SP) based on chains of modified polycarboxylic was used to improve the fresh state performance of SCC. Nominal particle sizes of 14 mm and 10 mm of coarse aggregate and well-graded sand were used in the mixture of SCC. The mix was optimized by the slump flow test, V-funnel test, and J-ring test aiming to achieve the requirements of filling ability, passing ability, and resistance to segregation according to the European guidelines for SCC. The compressive strength after 28 days was determined by a compressive test carried out on cubes measuring 100 mm × 100 mm × 100 mm.

BFRP is a new type of synthetic material, which is fabricated through the pultrusion molding process using basalt fiber as a reinforcement material and resin as a matrix material. BFRP bars have higher tensile strength and lower elastic modulus compared with steel bars (see Table 2). Besides the good mechanical properties, the BFRP reinforcement shows a high resistance to corrosion, and has a coefficient of thermal expansion similar to that of SCC. The average rupture strength of the BFRP bars was 920 MPa based on a loading rate of 0.2 kN/s, which is more appropriate for slab loading under a slow-moving vehicle and conservative compared to tests at a higher loading rate that gives higher rupture strength. The yield strength of steel reinforcement was obtained from the tensile test for reserved steel bar, and the average yield strength of the steel reinforcement was 520 N/mm^2^. The details of the material properties of the reinforcement are shown in Table 2.

### 2.3. Test Loading

**Figure 4:**
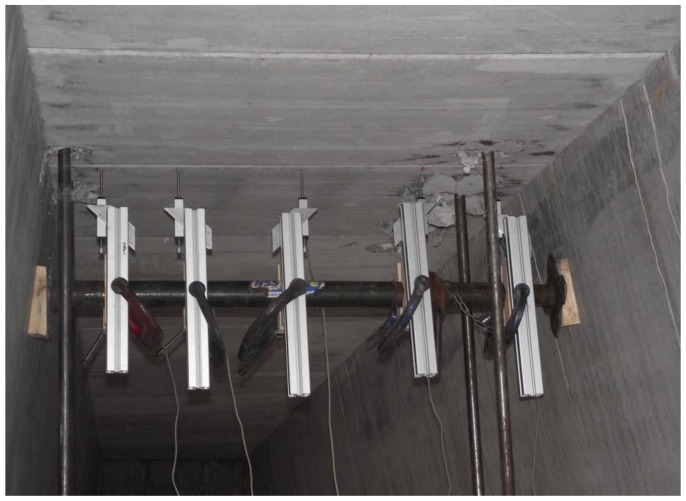
Position of the electronic displacement transducers.

**Figure 5:**
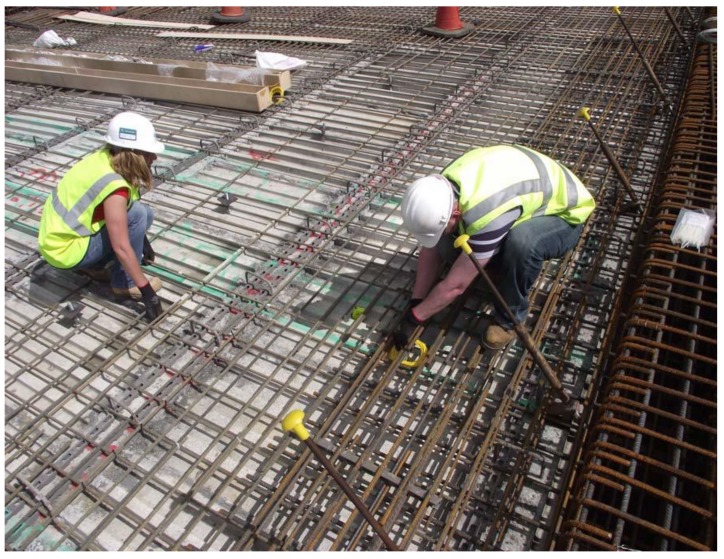
Positioning of the fiber bragg gratings (FBG) sensors.

The test program was designed to evaluate the influence of the reinforcement type and compressive membrane action on the structural behavior of the bridge deck slab. The test regions 1 to 4 of the bridge deck slab reinforced by BFRP had a reinforcement percentage of 0.6%, and the test regions 5 and 6 incorporated steel reinforcement with a reinforcement percentage of 0.7% (see Figure 2). As shown in Figure 4, five electronic displacement transducers were positioned along the center line of the loading area panel at midspan, quarter span, and near support. Figure 5 shows the Fiber Bragg Gratings (FBG) sensors installed on the reinforcement bar near the loading area for the purpose of monitoring the reinforcement strain.

**Figure 6:**
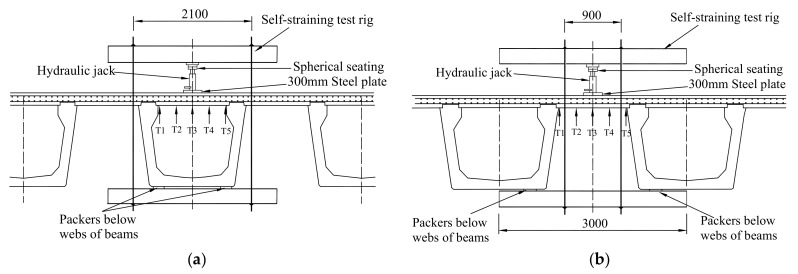
Test for the Thompson Bridge slab. (**a**) Test for the slab between the W-beams; (**b**) Test for the slab between W-beams and W-beams.

The effective span of the test regions 2, 3, and 5 between the W-beams and of the test regions 1, 4, and 6 between W-beams and W-beams are 1400 and 1600 mm, respectively. A typical test arrangement is shown in Figure 6. A circular concentrated load was applied on the test panel at midspan by a 300 mm diameter steel plate bedded on a soft board. An accurately calibrated 500 kN hydraulic jack system was applied to the load, and the test rig was assembled with the top beam horizontal to both axes in order to minimize eccentricity effects. A spherical seating was located between the ram and the loading beam to minimize the effect of any possible misalignment of the load.

All the test areas were loaded in increments of 5 kN, and the deflection and microstrain values were recorded at each increment. The bridge deck slabs were preloaded twice, in increments of 12.5 kN or 25 kN to 50 kN before the formal loading. A test load of over twice the current maximum wheel load (150 kN) given by Europe Code was applied incrementally in the formal loading. The deflection and microstrain values were recorded at each load increment.

### 2.4. Test Results and Analysis

**Figure 7:**
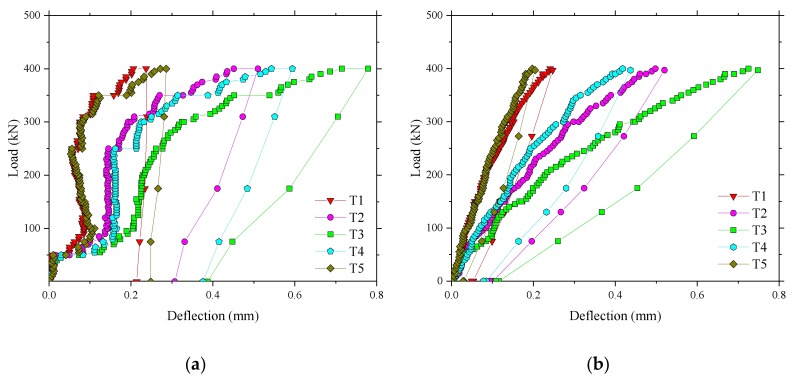
Deflection of different positions in the test areas. (**a**) Deflection for the test region 2; (**b**) Deflection for the test region 4.

Figure 7 illustrates the load–deflection relationship in the test regions of 2 and 4. The deflections at either side of the loaded area were virtually the same by comparing T1 and T5 and T2 andT4, which indicated very little eccentricity in the test arrangement. The deflection of the quarter span was about the average deflection of the near support and midspan at any load. An apparent slip is shown in Figure 7 a at a load of 50 kN, which is attributed to the instability of the acquisition instrument. There was a large residual deflection after unloading due to some problems with the jack device in unloading. However, 80% of the deflection was recovered after unloading in the test region 4. This implied that the Thompson Bridge was in its normal service loading when the load of 400 kN was applied.

**Figure 8:**
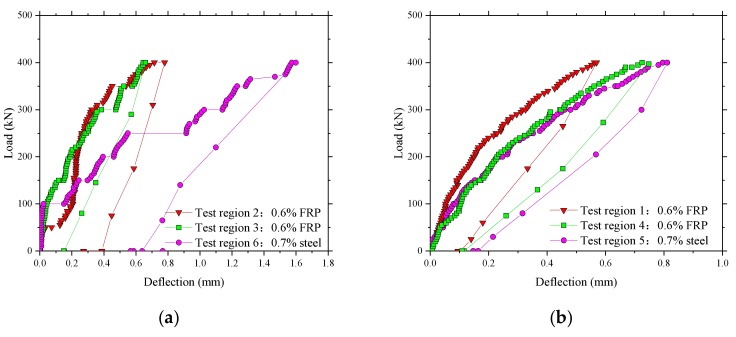
Comparison of midspan vertical deflections in the test areas. (**a**) The test areas between the W-beams; (**b**) The test areas between W-beams and W-beams.

The results of the maximum vertical deflections under full test load are compared in Figure 8a,b for the 1.6 m span slabs and 1.4 m span slabs, respectively. The deflections of all test panels were less than 2 mm at a maximum load of 400 kN. However, it can be seen that the test regions with steel reinforcement had higher deflections compared with those with BFRP reinforcement, especially in Figure 8a. This could be due to slight variations in the depth of the deck or to differences caused by concrete dispersion. The average deflection at the applied load of 150 kN, that is the European maximum wheel load [22], was 0.18 and 0.30 mm for the areas of BFRP reinforcement and steel in the 1.6 m span slabs, and 0.13 and 0.15 mm for the areas in the 1.4 m span slabs. This implicated that a very low deflection during the normal service state of Thompson Bridge will occur.

**Figure 9:**
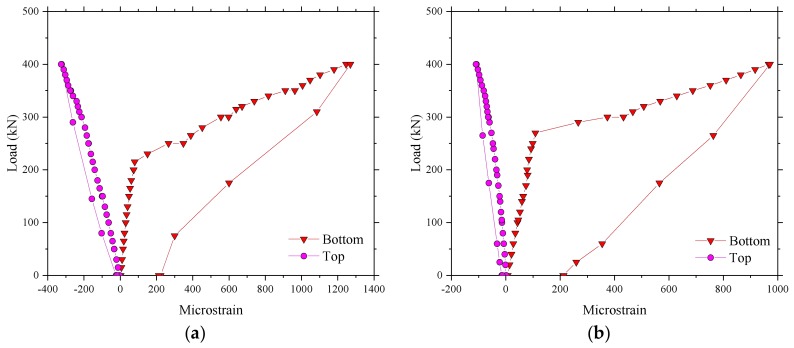
Microstrain in the test areas. (**a**) Microstrain for the test region 2; (**b**) Microstrain for the test region 1.

The results for the microstrain in the test regions 1 and 2 of the BFRP-reinforced deck slab are given in Figure 9. A similar load–strain curve appeared in the test regions 1 and 2, except that the strain in region 2 was larger than in region 1 at a maximum load of 400 kN. The maximum strains, as expected, were in the reinforcement bars of the bottom layer and were in tension (positive values). The maximum value of strain recorded was 1993 με in the test region 4. Table 2 shows that the ultimate strain of the BFRP bars based on the average values from tests on control samples was 17,037 με, and the maximum value of strain at an applied wheel load of 400 kN was 11.7% of the maximum possible strain for the BFRP bar. This represents a factor of safety of 8.5, nearly three times the European maximum wheel load [22]. A good performance of strain recovery for the sensors after unloading is shown in Figure 9.

## 3. Numerical Investigation of the Local Bridge Model

### 3.1. Nonlinear Finite Element Model

**Figure 10:**
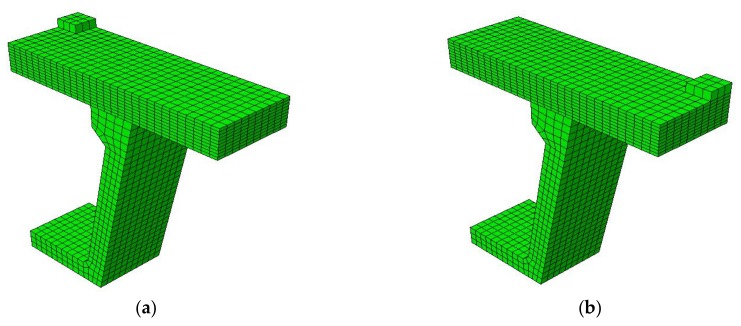
The finite element model for Thompson Bridge. (**a**) Loading for the slab between the W-beams in the finite element model; (**b**) Loading for the slab between W-beams and W-beams in the finite element model.

A commercial finite element analysis software named ABAQUS 6.10 was used as a tool of numerical investigation for failure mode and ultimate capacity of the Thompson Bridge deck slabs. It is accurate to predict the ultimate bearing capacity and failure mechanism of a FRP-reinforced concrete structure with the finite element method [8,23]. The bridge deck slabs and the supporting W-beams analyses were conducted using solid element owing to the stress distribution that can be clearly seen in the depth direction of the bridge deck, and eight-noded hexahedral (brick) reduced integration (C3D8R) elements were used for SCC to avoid the shear locking effect. In order to analyze the local behavior of the bridge deck under wheel load, the proposed finite element model of bridge slab supported by W-beam was built, as shown in Figure 10. Taking into account the test specimens’ symmetry and computational efficiency, a quarter-symmetric finite element model was used in this study.

In the finite element model, a truss element is adopted to simulate the reinforcement material inside the concrete structures. A bridge deck slab with lateral restraint has a perfect bond between bars and concrete under two-way reinforcement, and the bond-slip is generally not considered in the finite element model, so an embedded constraint was used in the bridge deck between the SCC and the reinforcement bars, in which the reinforcement material was designated as the embedded element, and the concrete was used as the host element. The loading surface of wheel load was modelled using a steel plate with the dimensions 300 mm × 300 mm × 60 mm. Because of the convergence difficulties in standard static solution of the FE analysis, a dynamic and explicit method was used for the numerical solution of the FE model in this study. To effectively simulate the descending section of the failure stage of the bridge slab, a method of displacement loading was adopted in the finite element model. An accurate result required that the mesh size depended on the maximum aggregate size of concrete, and the element size of the bridge slab in the thickness direction of 20 mm was adopted in the model.

### 3.2. Constitutive Models

#### 3.2.1. Damaged Plasticity Model

The model uses the yield function proposed by Lubliner et al. [24] and then modified by Lee and Fenves [25] to reflect the different evolution of strength under compression and tension, and the evolution of the yield surface is controlled by the hardening variables, compressive equivalent plastic strain (${\tilde{\epsilon }}_{c}^{\mathrm{pl}}$), and tensile equivalent plastic strain (${\tilde{\epsilon }}_{t}^{\mathrm{pl}}$). The yield function was defined according to Equation (1):

(1) $$F=\frac{1}{1-\alpha }\left(\bar{q}-3\alpha \bar{p}+\beta \left({\tilde{\epsilon }}^{\mathrm{pl}}\right)\langle {\bar{\hat{\sigma }}}_{\max}\rangle -\gamma \langle -{\bar{\hat{\sigma }}}_{\max}\rangle \right)-{\bar{\sigma }}_{c}\left({\tilde{\epsilon }}_{c}^{\mathrm{pl}}\right)$$

**Figure 11:**
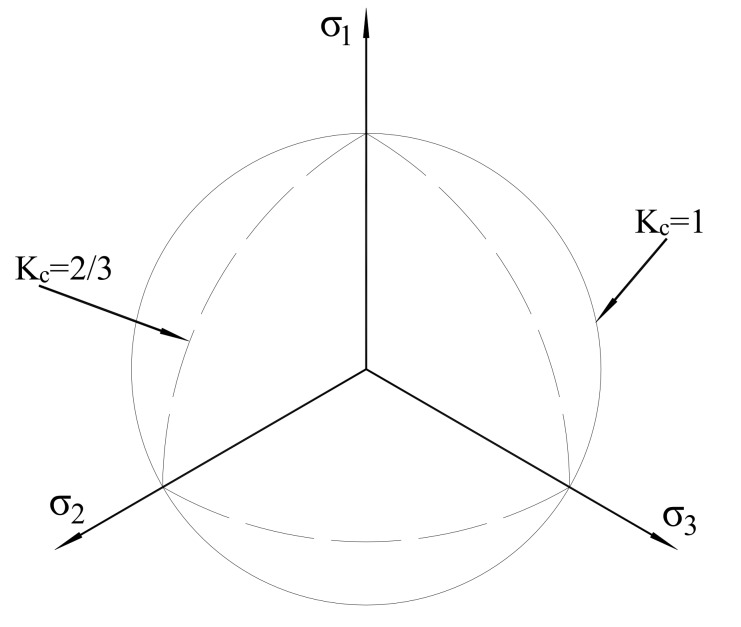
Yield surfaces in the deviatory plane.

The parameters *α*, *β*, and *γ* were calculated on the basis of Equations (2)–(4), respectively; $\bar{q}$ and $\bar{p}$ represent the Mises equivalent effective stress and the hydrostatic pressure stress, respectively; $\sigma_{b0}/\sigma_{c0}$ is the ratio of biaxial compressive yield stress to uniaxial compressive yield stress. The effective cohesion stresses for compressive and tensile status were expressed using ${\bar{\sigma }}_{c}\left({\tilde{\epsilon }}_{c}^{\mathrm{pl}}\right)$ and ${\bar{\sigma }}_{t}\left({\tilde{\epsilon }}_{t}^{\mathrm{pl}}\right)$*.* $K_{c}$ (the default value is 2/3) is the ratio of the second stress invariant on the tensile meridian to that on the compressive meridian, and the shape of yield surface was decided by $K_{c}$ in the deviatory plane (see Figure 11).

(2) $$\alpha =\frac{(\sigma_{b0}/\sigma_{c0})-1}{2(\sigma_{b0}/\sigma_{c0})-1};0\leq \alpha \leq 0.5$$

(3) $$\beta \left({\tilde{\epsilon }}^{\mathrm{pl}}\right)=\frac{{\bar{\sigma }}_{c}\left({\tilde{\epsilon }}_{c}^{\mathrm{pl}}\right)}{{\bar{\sigma }}_{t}\left({\tilde{\epsilon }}_{t}^{\mathrm{pl}}\right)}\left(1-\alpha \right)-\left(1+\alpha \right)$$

(4) $$\gamma =\frac{3\left(1-K_{c}\right)}{2K_{c}-1}$$

A nonassociate potential plastic flow was assumed, and the Drucker–Prager hyperbolic function was used as the flow potential G (see Equation (5)) in the damaged plasticity model. In order to ensure the unicity of flow direction, the flow potential was required to be continuous and smooth.

(5) $$G=\sqrt{{\left(\epsilon \sigma_{t0}\tan\psi \right)}^{2}+{\bar{q}}^{2}}-\bar{p}\tan\psi$$

where ψ is the dilation measured in the p−q plane at high confining pressure. The uniaxial tensile stress ($\sigma_{t0}$) at failure is obtained in the tensile test, and a parameter ϵ is defined as the rate at which the function approaches the asymptote; ϵ(=0.1) is the default flow potential eccentricity, which implies that the material has almost the same dilation angle over a wide range of confining pressure stress. If the value of ϵ is significantly less than the default value, there may be convergence problems when the material is subjected to a low confining pressure.

#### 3.2.2. Constitutive Relationship of Concrete

The constitutive relationship of concrete is complex and significant in the finite element model. Equation (6) shows the stress–strain relationship proposed by Saenz [26] for concrete under uniaxial compressive loading that was adopted in this study, where $\sigma_{p}$ and $\varepsilon_{p}$ represent the maximum stress and its corresponding strain, respectively; $\sigma_{p}$ and $\varepsilon_{p}$ are equal to the cylinder compressive strength ($f_{c}^{'}$) and 0.002, respectively, if the test data lacked [27].

(6) $$\sigma_{c}=\frac{E_{0}\varepsilon_{c}}{1+\left[\left(E_{0}\varepsilon_{p}/\sigma_{p}\right)-2\right]\left(\varepsilon_{c}/\varepsilon_{p}\right)+{\left(\varepsilon_{c}/\varepsilon_{p}\right)}^{2}}$$

The tension–softening curve of concrete was suggested by Hordijk [28] under uniaxial tension loading, and the relationship of stress–strain softening is shown in Equation (7) [29,30]:

(7) $$\frac{\sigma_{t}}{f_{t}}=\left[1+{\left(c_{1}\frac{\omega_{t}}{\omega_{\mathrm{cr}}}\right)}^{3}\right]e^{-c_{2}\frac{\omega_{t}}{\omega_{\mathrm{cr}}}}-\frac{\omega_{t}}{\omega_{\mathrm{cr}}}\left(1+c_{1}^{3}\right)e^{-c_{2}}$$

where $c_{1}$ = 3.0 and $c_{2}$ = 6.93, provided by the tensile test of concrete; $f_{t}$ and $\omega_{\mathrm{cr}}$ are the uniaxial tensile strength of concrete and crack opening displacement, respectively, when stress and energy were released completely. The $\sigma_{t}$ and $\omega_{t}$ represent the tension stress normal to the crack direction and the crack open displacement, respectively.

**Figure 12:**
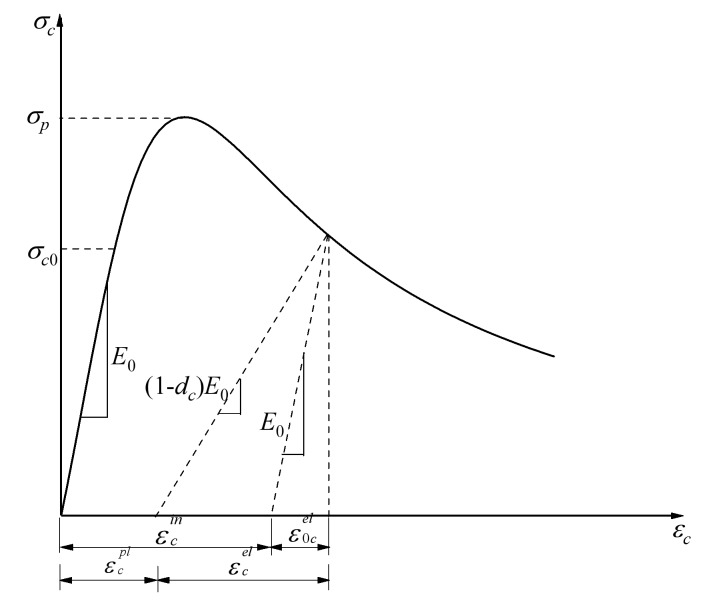
Response of concrete to uniaxial loading in compression.

**Figure 13:**
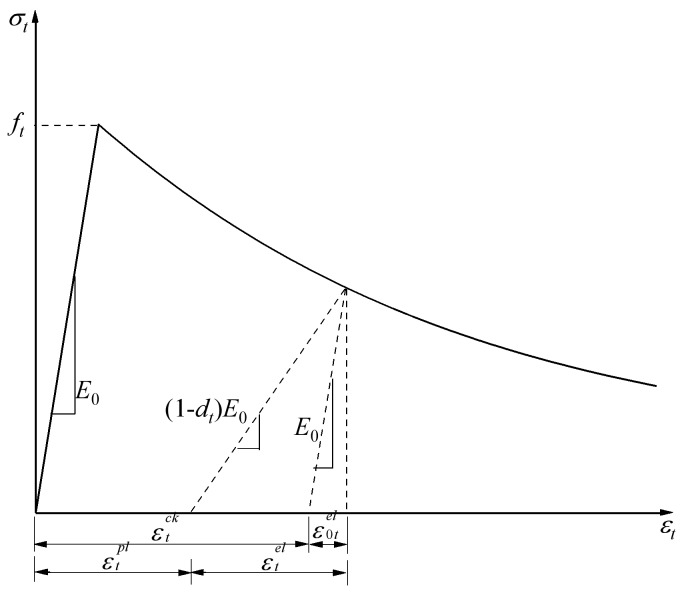
Response of concrete to uniaxial loading in tension.

Figure 12 and Figure 13 show the response of compression and tension in uniaxial loading for concrete, respectively. Concrete appears to be linearly elastic before reaching the initial yield stress $\sigma_{c0}$, which is followed by a strengthening stage until the ultimate stress $\sigma_{p}$ is reached and then a softening stage in uniaxial compression loading. There is no strengthening stage in uniaxial tensile loading compared with uniaxial compression loading; ${\tilde{\varepsilon }}_{c}^{\mathrm{in}}$ and ${\tilde{\varepsilon }}_{t}^{\mathrm{in}}$ were applied in the finite element model as hardening data.

The reduction of the elastic modulus was assumed in the concrete damaged plasticity model, and a scalar degradation variable d was shown in Equation (8) to reflect the concrete material behavior in the inelastic range, where $E_{0}$ is the undamaged modulus of the concrete material.

(8) $$E=\left(1-d\right)E_{0}$$

The tensile cracking and compressive crushing are the main failure mechanisms of concrete in the concrete damaged plasticity model. The stiffness degradation variable d is the function of the stress state, and the uniaxial damage variables are $d_{c}$ and $d_{t}$ (see Equation (9)); $s_{t}$ and $s_{c}$ represent the stiffness recovery of tension and compression:

(9) $$\left(1-d\right)=\left(1-s_{t}d_{c}\right)\left(1-s_{c}d_{t}\right)$$

Equations (10) and (11) can be deduced from the stress–strain relationships of compression and tension (see Figure 12 and Figure 13), respectively:

(10) $$\sigma_{c}=\left(1-d_{c}\right)E_{0}\left(\varepsilon_{c}-{\tilde{\varepsilon }}_{c}^{\mathrm{pl}}\right)$$

(11) $$\sigma_{t}=\left(1-d_{t}\right)E_{0}\left(\varepsilon_{t}-{\tilde{\varepsilon }}_{t}^{\mathrm{pl}}\right)$$

By substituting $\varepsilon_{c}={\tilde{\varepsilon }}_{c}^{\mathrm{in}}+\varepsilon_{0c}^{\mathrm{el}}$, $\varepsilon_{0c}^{\mathrm{el}}=\sigma_{c}E_{0}^{-1}$ into Equation (10) and $\varepsilon_{t}={\tilde{\varepsilon }}_{t}^{\mathrm{ck}}+\varepsilon_{0t}^{\mathrm{el}}$, $\varepsilon_{0t}^{\mathrm{el}}=\sigma_{t}E_{0}^{-1}$ into Equation (11), uniaxial damage variables can be expressed as:

(12) $$d_{c}=1-\frac{\sigma_{c}E_{0}^{-1}}{{\tilde{\varepsilon }}_{c}^{\mathrm{pl}}\left(1/b_{c}-1\right)+\sigma_{c}E_{0}^{-1}}$$

(13) $$d_{t}=1-\frac{\sigma_{t}E_{0}^{-1}}{{\tilde{\varepsilon }}_{t}^{\mathrm{pl}}\left(1/b_{t}-1\right)+\sigma_{t}E_{0}^{-1}}$$

where $b_{c}={\tilde{\varepsilon }}_{c}^{\mathrm{pl}}/{\tilde{\varepsilon }}_{c}^{\mathrm{in}}$, $b_{t}={\tilde{\varepsilon }}_{t}^{\mathrm{pl}}/{\tilde{\varepsilon }}_{t}^{\mathrm{ck}}$. $b_{c}=0.7$, and $b_{t}=0.1$ were recommended by the stress path in the calibration of the unloaded state by cyclic loading [31].

#### 3.2.3. FRP and Steel Reinforcement

**Figure 14:**
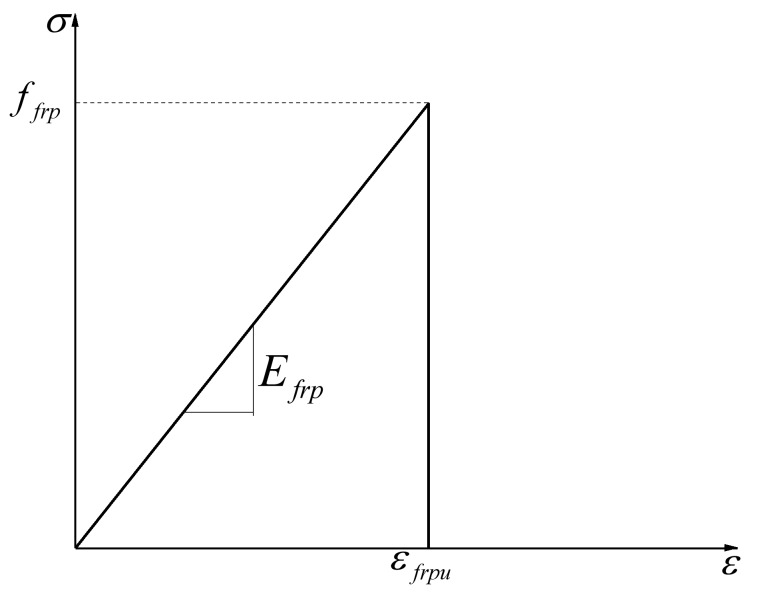
Constitutive relationship of fiber-reinforced polymer (FRP) reinforcement.

**Figure 15:**
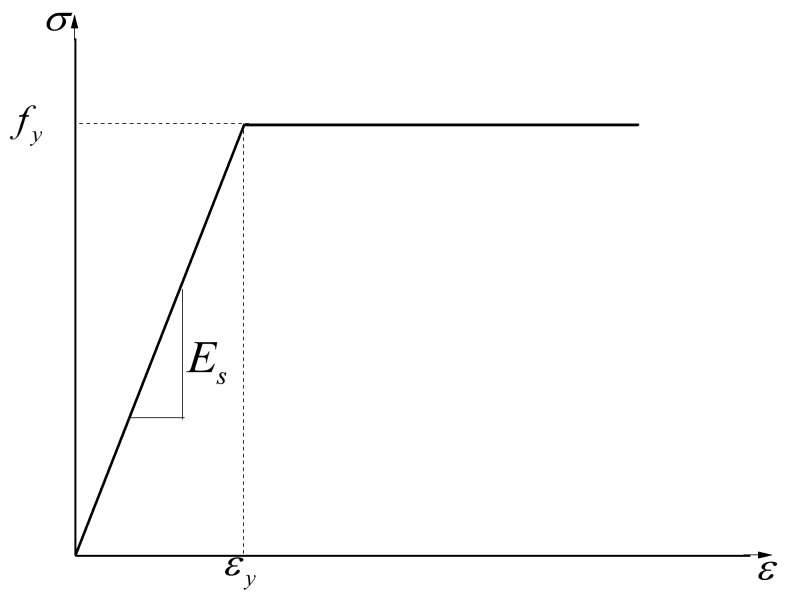
Constitutive relationship of steel reinforcement.

The stress–strain relationships of FRP and steel reinforcement are represented in Figure 14 and Figure 15, respectively. FRP reinforcement is ideal in linear elastic materials until a brittle damage occur when the ultimate tensile stress ($f_{frp}$) is reached. The Linear-elastic brittle model and the elastic perfectly plastic model were adopted for FRP reinforcement and steel reinforcement in the finite element model, respectively. The material properties of the reinforcement for numerical simulation are shown in Table 3; the parameters of the reinforcement were provided by the manufacturer or were determined by experimental tests.

### 3.3. Validation of the Accuracy of the FE Analsysis

**Figure 16:**
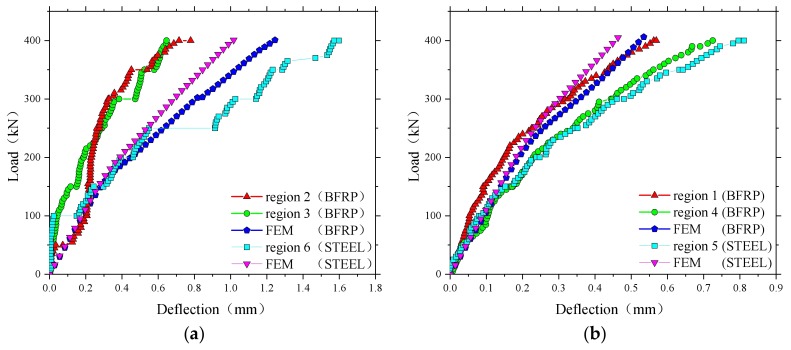
Comparison of load–displacement responses obtained from the experimental test and the finite element method (FEM). (**a**) Loading between the W-beams; (**b**) Loading between W-beams and W-beams.

The comparison between the load–deflection displacement responses of the bridge deck slabs from the experimental test and the numerical analysis are shown in Figure 16. It can be seen in the numerical analysis that the steel-reinforced bridge deck slab had a smaller deflection compared to the BFRP-reinforced bridge deck at the same load level. The reason for this phenomenon is the stiffness of the steel reinforcement which is larger than that of the BFRP reinforcement [32]. In general, the load–deflection curve of the deck slabs from the numerical results had a good correlation with the experimental test results. However, Figure 16 shows that the predicted structural response in the proposed FE model is a bit stiffer than that in the field experimental tests. This stiffer phenomenon is also shown in the author’s previous FE investigation of concrete bridge deck slabs [13]. This could be due to the major drawback of the smeared crack assumption used in this simulation, which is the inability to model the surface of the crack [33]. In addition, the representation of smeared cracked materials as a continuum induces locked-in stress in the elements close to the localization zone [34]. It was found that the stiffness of the numerical model for bridge deck slabs decreased when the load reached 150 kN, which implies that no or little damage in the numerical model had occurred when the European maximum wheel load (150 kN) was applied [35].

**Figure 17:**
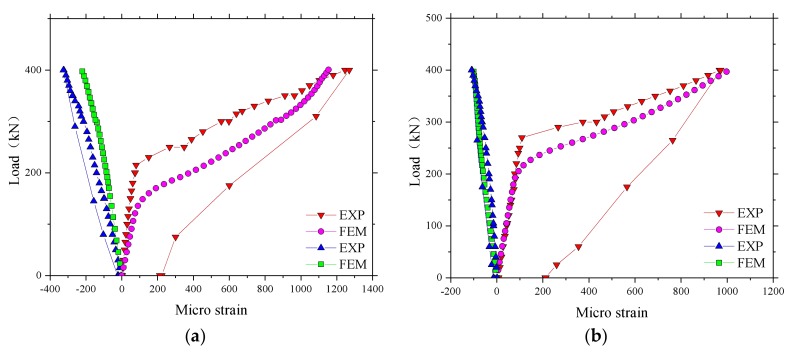
Comparison of load–strain responses obtained from the experimental test and the FEM. (**a**) Loading between the W-beams; (**b**) Loading between W-beams and W-beams.

Figure 17 presents the comparison of load–microstrain responses obtained from the experimental test and the FEM for the BFRP reinforcement. It was found that the load–microstrain curve of the numerical results for the bridge deck slabs showed good agreement with the experimental test results. The microstrain of the BFRP reinforcement significantly increased in the experimental test when the loading of 250 kN was reached. However, a lower load was found in the numerical results compared with 250 kN when the microstrain significantly increased. The maximum value of microstrain at an applied wheel load of 400 kN was relatively smaller compared with the maximum possible strain of the BFRP bar, whether in the experimental test results or in the numerical results. This indicates that the BFRP reinforcement did not have a strong effect when the bridge deck slabs were in the normal service state, which is due to the CMA inside the bridge deck slabs with high laterally restraint stiffness.

On the basis of the validation numerical analysis results, it can be concluded that the proposed finite element model is suitable for analyzing the structural behavior and failure mechanism of bridge deck slabs in the study. These finding are discussed in the following sections.

### 3.4. Discussion of the Stress Distribution

**Figure 18:**
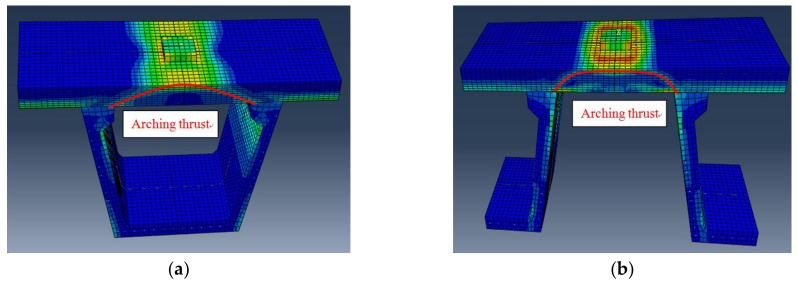
Von Mises stress clouds of the finite element model. (**a**) Von Mises stress cloud for slab loading between the W-beams; (**b**) Von Mises stress cloud for slab loading between W-beams and W-beams.

The Von Mises stress clouds of bridge deck slabs are shown in Figure 18. It can be seen that the Von Mises stress distribution of the finite element model can accurately simulate the compressive membrane action inside the concrete bridge deck slabs, and the arch thrust line can be clearly seen from the Von Mises stress distribution. This compressive membrane action was caused by the strong laterally restraint stiffness provided by the supporting W-beams.

**Figure 19:**
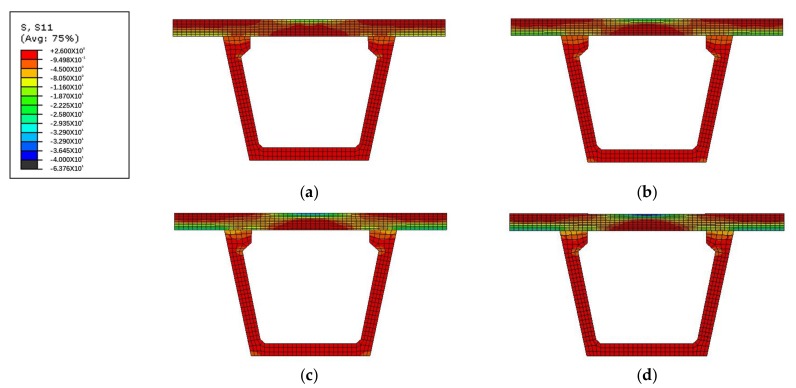
Transverse stress distribution for slab loading between the W-beams. (**a**) At 50% of the ultimate bearing capacity; (**b**) At 70% of the ultimate bearing capacity; (**c**) At 90% of the ultimate bearing capacity; (**d**) At 100% of the ultimate bearing capacity.

Figure 19 presents the transverse stress developing from 50% of the ultimate bearing capacity to 100% of the ultimate bearing capacity of concrete bridge deck slabs through the depth for the finite element model. From the transverse stress distribution in the slab numerical analysis, it was found that, at 50% of the ultimate bearing capacity value, the highest transverse compressive stress occurred in the loading area on the top surface of the bridge deck slab, while the tensile stress developed in the loading area on the bottom and top surfaces of the supporting W-beam. At 70% of the ultimate bearing capacity value, the compressive stress on the top surface of the loading area and the bottom of the supporting W-beam increased rapidly with further application of the load, the transverse compressive stress was more significant between the loading zone and the W-beam support region, and compressive membrane thrust further developed. At 90% of the ultimate bearing capacity value, an arc-shaped arch thrust line was perfectly displayed on the side of the bridge deck slab, which indicated that the compressive membrane action had fully developed. When the ultimate bearing capacity was applied, the compressive stress on the top surface of the loading areas reached the ultimate compressive stress, which induced crushing in the concrete deck slabs.

### 3.5. Prediction of the Ultimate Bearing Capacity and Failure Mechanism

**Figure 20:**
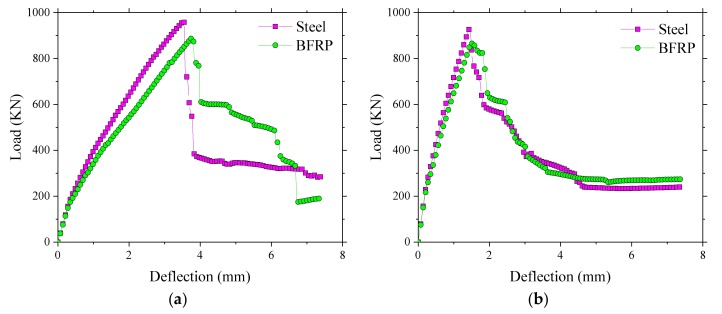
Load–deflection response in deck slabs by finite element (FE) analysis. (**a**) Load–deflection curve between the W-beams in the finite element model; (**b**) Load–deflection curve between W-beams and W-beams in the finite element model.

The ultimate bearing capacity of the SCC deck slabs in Thompson Bridge was predicted (see Figure 20) by the finite element model. It can be seen that the ultimate load-carrying capacity, about 1000 kN, was basically the same for loading between the W-beams and loading between W-beams and W-beams. The load–deflection curve of the SCC deck slabs with BFRP rebar reinforcement in Thompson Bridge provided a similar ultimate behavior compared with steel-reinforced SCC deck slabs. The ultimate load at a reinforcement ratio of 0.6% was more than six times the maximum wheel load given by Europe Code, that is 150 kN [35]. This indicates that there was a large ultimate bearing capacity at low percentage of reinforcement, which may be attributed to the arching action in the bridge deck slabs. In addition, it was shown that the reinforcing material type does not have a strong effect on the loading-carrying capacity in laterally restrained SCC deck slabs [19].

**Figure 21:**
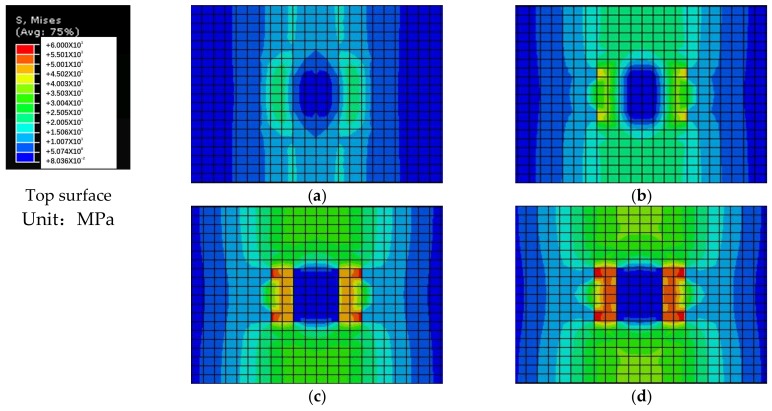

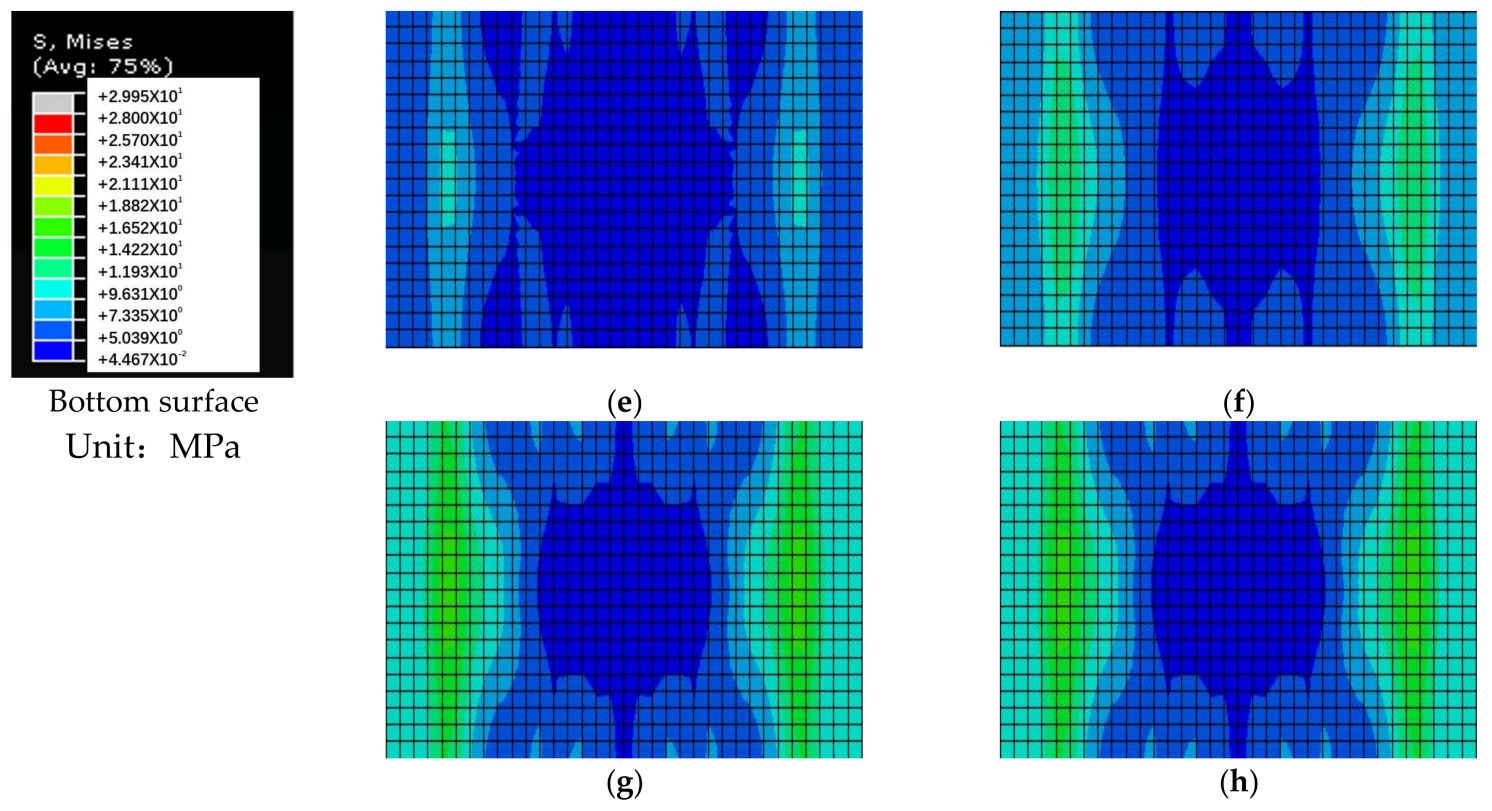
Von Mises stress distribution on the top and bottom surfaces between the W-beams. (**a**) At 50% of the ultimate bearing capacity; (**b**) At 70%; (**c**) At 90%; (**d**) At 100%; (**e**) At 50%; (**f**) At 70%; (**g**) At 90%; (**h**) At 100%.

Figure 21 demonstrates the distribution of the Von Mises stress from 50% of the ultimate bearing capacity to 100% of the ultimate bearing capacity on the top and bottom surfaces in concrete bridge deck slabs for the finite element model. On the top surface, the Von Mises stress increased to extend into the W-beam supporting locations when the load was enhanced from 50% of the ultimate bearing capacity to 70% of the ultimate bearing capacity. At 90% of the ultimate bearing capacity, the highest Von Mises stress appeared near the loading area, and the stress extended further toward the W-beam support. Until the ultimate bearing capacity was reached, the stress near the loading zone exceeded the ultimate stress, and the top concrete was crushed. On the bottom surface, from 50% of the ultimate bearing capacity to 70% of the ultimate bearing capacity, the stress of the W-beam supporting position was increased and extended to the center loading zone. When the applied load reached 90% of the ultimate bearing capacity or more, the tension zone was significantly regionalized, and this could cause snap-through failure until the loss of the bearing capacity. According to the analysis of the Von Mises stress obtained from the finite element model, it can be inferred that, as a consequence of the arching action, the failure mode of the bridge deck slabs under a wheel load could commonly be the punching failure, which was also investigated by some Canadian researchers [36,37].

## 4. Parameter Analysis of the Local Bridge Model

In this parametric study through FE analysis, four structural variables, namely, reinforcement ratio, reinforcement type, concrete compressive strength, and depth of bridge deck slab, were involved. The influence of the four structural variables on the ultimate state of the bridge deck slab will be discussed separately.

### 4.1. Effect of the Reinforcement Ratio

**Figure 22:**
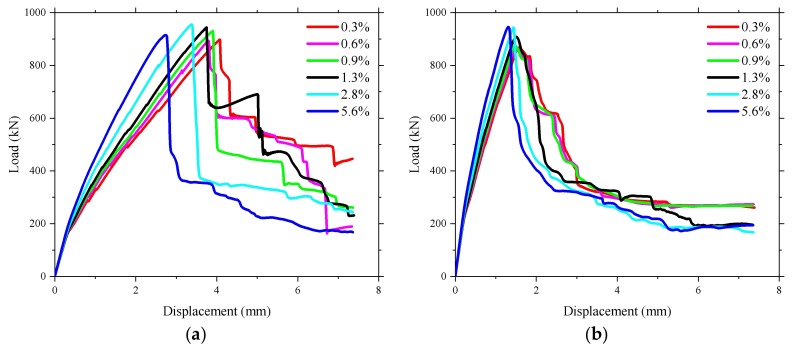
Influence of the reinforcement ratio on the ultimate bearing capacity. (**a**) Loading between the W-beams; (**b**) Loading between W-beams and W-beams.

**Figure 23:**
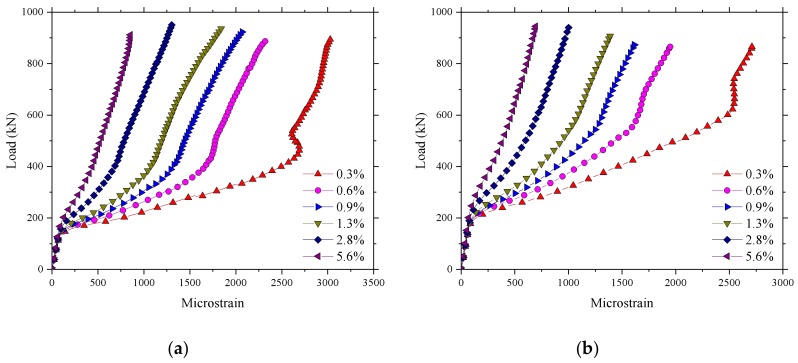
Influence of the reinforcement ratio on the strain of reinforcement. (**a**) Loading between the W-beams; (**b**) Loading between W-beams and W-beams.

Figure 22 shows that there was no significant change in the ultimate bearing capacity as the reinforcement ratio increased, which could be attributed to the high tensile properties of reinforcement being not fully utilized because of the existence of the compressive membrane action. The results of the finite element indicate that the increase of the reinforcement ratio had no influence on the cracking load of the bridge deck slab. However, the stiffness after cracking increased, and the displacement under the ultimate load decreased with the increase of the reinforcement ratio. The maximum microstrain that appeared near the loading area is shown in Figure 23. It can be seen that the microstrain increased significantly after cracking, and the maximum microstrain decreased with the increase of the reinforcement ratio. However, the ultimate microstrain at a reinforcement percentage of 0.3% was about 3000 με, which is 17.6% of the maximum possible strain of 17,037 με. This implies that the presence of the compressive membrane action restricted the effect of the reinforcement on the bridge deck, and very low percentage of reinforcement can be used in bridge deck slabs by using the benefits of the arching action.

### 4.2. Effect of the Reinforcement Type

**Figure 24:**
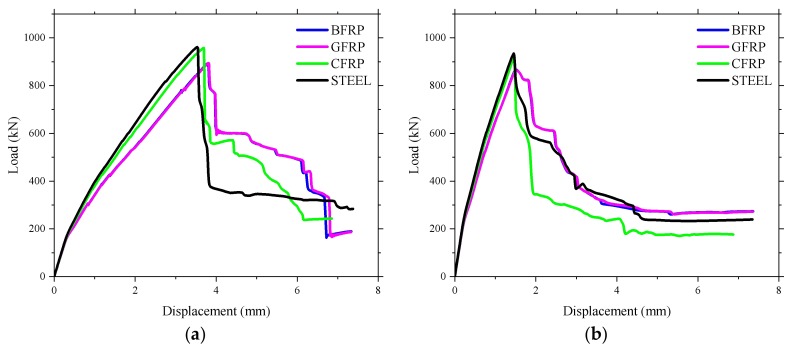
Influence of the reinforcement type on the ultimate bearing capacity. (**a**) Loading between the W-beams; (**b**) Loading between W-beams and W-beams.

The effects of different types of reinforcement, such as Glass Fiber Reinforced Polymer (GFRP) rebar, BFRP rebar, Carbon Fiber Reinforced Polymer (CFRP) rebar, and steel rebar, on the structural performance of the concrete deck slabs in the numerical simulation is presented in Figure 24. The material properties of different types of reinforcement set in the finite element model are shown in Table 3. The axial stiffness and axial force of different reinforcement types are shown in Table 4.

**Figure 25:**
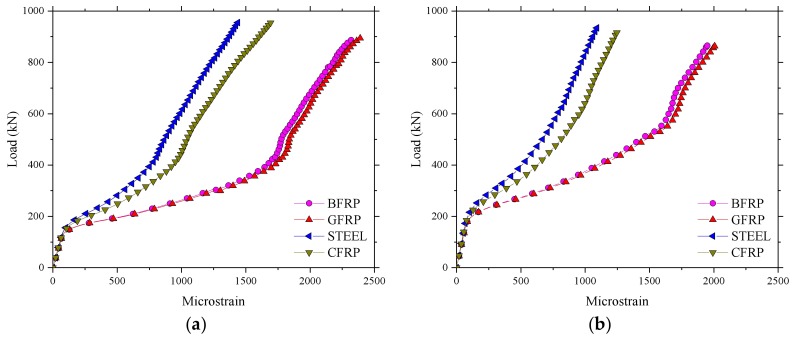
Influence of the reinforcement type on the strain of reinforcement. (**a**) Loading between the W-beams; (**b**) Loading between W-beams and W-beams.

Figure 24 demonstrates that the reinforcement type had a little influence on the cracking load and the ultimate bearing capacity. However, the stiffness of the bridge deck slab was different after cracking, which could be related to the different axial stiffnesses of the reinforcing materials. Figure 25 shows the relationship between microstrain and load of the bridge deck slab reinforced with different reinforcement types. It was found that the microstrain of the reinforcement increased sharply after concrete cracking. However, it can be noted that varying the reinforcing materials with the same reinforcement percentage did not have a strong effect on the structural behavior of the laterally restrained deck slabs, which was due to the contribution of the compressive membrane action. The structural stiffness of concrete deck slabs reinforced with CFRP and steel bars were slightly higher than those of the deck slabs reinforced with BFRP and CFRP, which was due to the different elastic moduli of the reinforcements. In addition, increasing the axial stiffness of the reinforcement resulted in reduced strain inside the reinforcing bars, as shown in Figure 25. This indicates that the contribution of the reinforcement materials decreased by increasing the axial stiffness of the reinforcement in lateral restrained concrete deck slabs.

### 4.3. Effect of Concrete Compressive Strength

**Figure 26:**
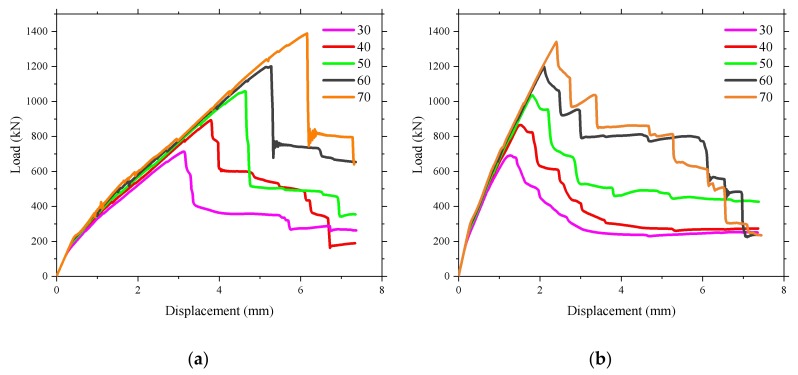
Influence of concrete compressive strength on the ultimate bearing capacity. (**a**) Loading between the W-beams; (**b**) Loading between W-beams and W-beams.

**Figure 27:**
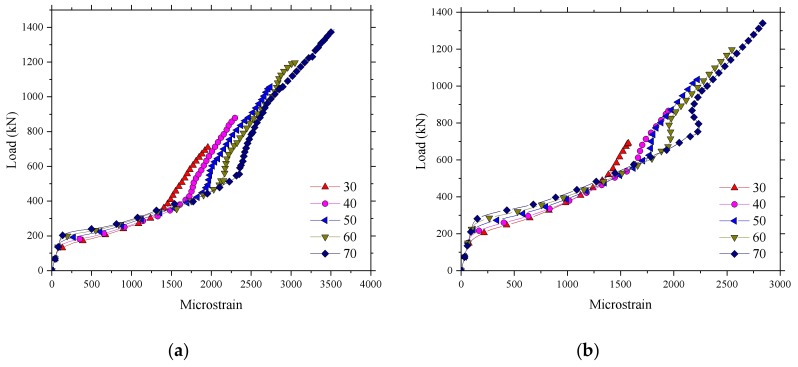
Influence of concrete compressive strength on the strain of the reinforcement. (**a**) Loading between the W-beams; (**b**) Loading between W-beams and W-beams.

Figure 26 presents the influence of concrete compressive strength on the ultimate bearing capacity. It can be seen that the ultimate load of the BFRP-reinforced SCC deck slabs increased by an average of about 165 kN due to an increased concrete strength of 10 MPa. Additionally, it was also found that the cracking load and stiffness after cracking were enhanced slightly by increasing the concrete strength (Figure 26 and Figure 27). This was due to the improvement of the tensile strength with the increase in the concrete strength. There are two inflection points in Figure 27: the first inflection point is ascribed to stiffness degradation of the bridge deck structure after cracking of the concrete, and the second inflection point could be attributed to the compressive membrane action. After the first inflection point appeared, the strain of the reinforced material increased rapidly; however, the increase rate of the strain decreased after the second inflection point.

### 4.4. Effect of the Depth of the Bridge Deck Slab

**Figure 28:**
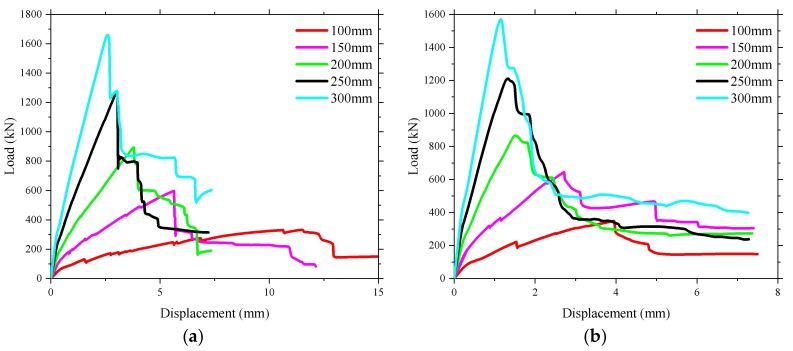
Influence of the depth of the bridge deck slab on the ultimate bearing capacity. (**a**) Loading between the W-beams; (**b**) Loading between W-beams and W-beams.

**Figure 29:**
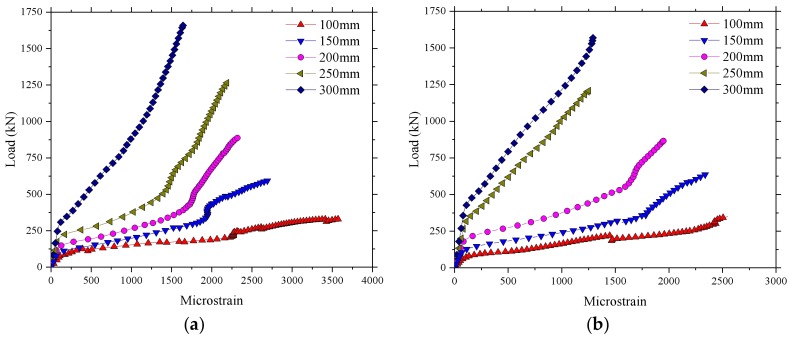
Influence of the depth of the bridge deck slab on the strain of the reinforcement. (**a**) Loading between the W-beams; (**b**) Loading between W-beams and W-beams.

The influence of the depth of the bridge deck slab on the ultimate bearing capacity was investigated (see Figure 28). It was found that increasing the depth of the bridge deck slab provided a significant enhancement in the ultimate load and cracking load. A 50% increase in the depth of the bridge deck slab resulted in an average increase in the ultimate load of approximately 35% in the numerical analysis prediction. The stiffness after cracking of the bridge deck slab increased significantly, and the displacement under the ultimate load decreased because of the correspondent increase of the bridge deck slab depth. The increasing of the depth of the bridge deck slab resulted in the reduction of the microstrain of the reinforcement at the same load level (see Figure 29).

## 5. Conclusions

This paper reveals an FE study of the structural behavior of BFRP-reinforced SCC deck slabs under wheel loads in a real bridge, namely, the Thompson Bridge. From this study, the following conclusions can be drawn:

1. The results of the field test in Thompson Bridge showed that the deflections and strain values of BFRP-reinforced concrete deck slabs under a wheel load of 400 kN were within an acceptable service range. However, it is worth mentioning that BFRP-reinforced SCC deck slabs exhibited better structural behaviors that that is predicted by current design codes, due to the contribution of the compressive membrane action to the structural behaviors of restrained deck slabs.
2. The results of the proposed FE model for BFRP-reinforced SCC deck slabs in Thompson Bridge showed good agreement with the field test results.
3. The FE analysis results indicated that BFRP-reinforced SCC deck slabs used in Thompson Bridge exhibited Compressive Membrane Action (CMA), as clearly obtained in the FE analysis.
4. The ultimate load-carrying capacity of the SCC deck slabs with BFRP-reinforced was predicted to be approximately 1000 kN by the FE model, which far exceeds the design ultimate loads. Because of the influence of CMA, the SCC deck slabs reinforced with BFRP bars had a similar reinforcement percentage as those reinforced with steel bars. In addition, the predicted failure mode of the SCC deck slabs reinforced with BFRP bars under wheel load was determined to be the punching failure by the FE model.
5. The parametric analysis by the FE model demonstrated that the reinforcement ratio and reinforcement type have an insufficient effect on the structural behavior of the bridge deck slabs. This is attributed to the contribution of CMA inside the laterally restrained deck slabs. However, the concrete compressive strength and the depth of the bridge deck slabs play a significant role in the structural mechanical properties of the bridge deck slabs under ultimate and service state.
6. The FE investigation results indicates that the BFRP-reinforced SCC deck slabs showed similar structural behavior as the steel-reinforced normal concrete (NC) deck slabs in terms of compressive membrane action. However, because of the use of a high volume of an industrial by-product (fly ash) in the SCC mixture of this study, the sustainability and durability of SCC can be improved compared to traditional NC. Additionally, using the benefits of the compressive membrane action it is possible to configure a very low reinforcement ratio of BFRP bars in concrete deck slabs. On the basis of the corrosion-free property of the BFRP bars, these findings indicate that the combination of the compressive membrane action, BFRP, and SCC can produce low-carbon footprint, durable, and economical infrastructures.

## Figures and Tables

**Table 1:** Self-compacting concrete (SCC) material properties.

Test	Slump Flow (mm)	V-Funnel (s)	J-Ring (mm)	Compressive Strength (MPa)
Test Result	650	12	8	50.5

**Table 2:** Reinforcement bar material properties. BRFP, basalt fiber-reinforced polymer

Type	Tensile Strength (MPa)	Yield Strength (MPa)	Elastic Modulus (MPa)	Ultimate Strain (με)
BFRP bars	920	/	54,000	17,037
Steel bars	750	520	210,000	10,000

**Table 3:** Material properties of the reinforcements.

Type	Density (kg/m^3^)	Elastic Modulus (MPa)	Poisson’s Ratio	Tensile Strength (MPa)	Ultimate Strain (με)
BFRP Bar	1900	54,000	0.2	920	17,037
GFRP Bar	2000	51,000	0.2	1610	31,000
CFRP Bar	1500	150,000	0.2	1700	11,000
Steel Bar	7800	210,000	0.3	520 (750)	10,000

Note: 520 MPa is the yield strength, and 750 MPa is the ultimate strength of steel bars. Notation: BFRP- Basalt Fiber Reinforced Polymer; GFRP-Glass Fiber Reinforced Polymer; CFRP-Carbon Reinforced Polymer.

**Table 4:** Mechanical properties of different types of reinforcement.

Type	Cross Section of Bars (m^2^)	Elastic Modulus (MPa)	A*E (m^2^·MPa)	Tensile Strength (MPa)	A*f(m^2^·MPa)
BFRP Bar	A	54,000	54,000 A	920	920 A
GFRP Bar	A	51,000	51,000 A	1610	1510 A
CFRP Bar	A	150,000	150,000 A	1700	1700 A
Steel Bar	A	210,000	210,000 A	750	750 A

Note: “A” represents the total area of longitudinal tension reinforcement. The longitudinal reinforcement ratio of different reinforcement type is 0.6%.
